# Development and validation of a predictive model for chronic pain after thoracoscopic pulmonary resection

**DOI:** 10.3389/fpubh.2026.1787875

**Published:** 2026-06-19

**Authors:** Yue Shang, Chenyang Wang, Jiameng Wang, Wenlun Zhong, Changjun Gao

**Affiliations:** 1 Department of Anesthesiology, Tangdu Hospital, The Fourth Military Medical University, Xi'an, China; 2 Graduate School, Yan'an University, Yan'an, Shaanxi, China; 3 Graduate School, Xi'an Medical University, Xi'an, Shaanxi, China

**Keywords:** associated predictors, chronic postsurgical pain, multimodal analgesia, predictive model, thoracoscopic pulmonary resection

## Abstract

**Background:**

Chronic postsurgical pain (CPSP) is a major complication following video-assisted thoracoscopic surgery (VATS) for lung resection. Despite its clinical importance, the reported incidence of CPSP varies considerably across studies, and a standardized tool for risk prediction remains lacking. This study aimed to identify predictors associated with CPSP after VATS lung resection within a single-center prospective observational cohort and to develop predictive models to guide personalized analgesic strategies.

**Methods:**

Clinical data were analyzed from patients who underwent thoracoscopic pulmonary resection at Tangdu Hospital between August 2024 and July 2025. Eligible patients were randomly allocated into a training cohort (80%) for model development and an internal validation cohort (20%) for model validation. Acute pain was assessed using the highest Numerical Rating Scale (NRS) score recorded within 72 h postoperatively, while CPSP was evaluated at 3 months after surgery. Patients were dichotomized based on the presence of CPSP into the CPSP group and the non-CPSP group. Variables with a *P*-value <0.1 in univariate analysis were entered into multivariate logistic regression to establish a prediction model for CPSP using R software.

**Results:**

A total of 744 patients were included in this study, with 595 assigned to the training set and 149 to the validation set. Among all patients, 284 (38.1%) developed CPSP. The incidence rates in the training and validation cohorts were 37.8 and 36.9%, respectively. Multivariate logistic regression identified gender, postoperative acute pain, postoperative pneumonia, duration of chest tube drainage, and opioid rescue dose (in morphine milligram equivalents) as independent predictors associated with CPSP. The nomogram model constructed based on these predictors demonstrated good performance, with an area under the curve (AUC) of 0.880 (95% CI: 0.850–0.910). Calibration curve analysis confirmed the high predictive accuracy of the model (Hosmer–Lemeshow test, *P* = 0.737), and decision curve analysis indicated a significant clinical net benefit. Furthermore, in the validation cohort, the model maintained good discriminative ability (AUC = 0.829) and calibration (Hosmer–Lemeshow test, *P* = 0.765), enabling reliable individualized risk prediction.

**Conclusions:**

CPSP remains highly prevalent after thoracoscopic pulmonary resection.

## 1 Introduction

The global incidence of lung cancer continues to rise annually. According to recent statistics, lung cancer was the leading cause of cancer-related morbidity and mortality worldwide in 2022, accounting for nearly 2.5 million new cases (12.4% of all cancers) and approximately 1.8 million deaths (18.7% of all cancer deaths), representing nearly 15% of total cancer-related mortality (1). Common treatment modalities for lung cancer include surgical resection, drug therapy, and interventional therapy, with surgical resection remaining the preferred option for early-stage disease. Moreover, with advancements in thoracoscopic techniques for lung cancer surgery, the clinical survival rates of many patients have significantly improved.

However, associated complications also warrant attention. Among these, chronic postsurgical pain (CPSP) represents a significant concern that cannot be overlooked. Long-term CPSP not only diminishes patients' quality of life but may also predispose to additional complications. Postoperative acute pain refers to pain that occurs within 24–72 h following surgical stimulation (2). CPSP is clinically defined as persistent pain that develops or intensifies at the surgical site or its surrounding area, lasting for more than 3 months after a surgical procedure or tissue injury (3). Compared with thoracotomy, video-assisted thoracoscopic surgery (VATS) substantially reduces postoperative pain, shortens recovery time, and improves quality of life (4). Nevertheless, studies have reported that the incidence of chronic pain after thoracoscopic surgery can still range from 27.1 to 43.5% (5).

The incidence and risk factors of chronic pain after thoracoscopic lung resection have long been a clinical concern for anesthesiologists. Studies have identified several risk factors for chronic pain following thoracoscopic pulmonary resection, including female sex, age ≤ 60 years, obesity, sleep disturbances, psychological factors, traumatic stimuli, surgical duration exceeding 3 h, multi-port thoracoscopic procedures, prolonged placement of a thoracic drainage tube, and postoperative acute pain (6–8). Among these, postoperative acute pain is the most significant contributor to CPSP. However, studies on the development of risk prediction models for chronic pain after thoracoscopic lung resection remain scarce. Due to variations in follow-up duration and inconsistencies in pain assessment scales, the reported incidence of thoracoscopic CPSP varies widely, leading to heterogeneity among existing prediction models (9). Furthermore, many studies have failed to thoroughly investigate how to predict and manage CPSP, largely owing to individual patient differences and the absence of standardized treatment protocols.

In summary, this study aims to investigate the incidence and associated predictors of CPSP in patients undergoing thoracoscopic pulmonary resection and to develop a prediction model. This model is intended to facilitate individualized interventions for high-risk patients and enable timely adjustments to perioperative pain management strategies, thereby reducing the incidence of chronic pain following thoracoscopic surgical procedures.

## 2 Methods

### 2.1 Research subjects

This study was approved by the Ethics Committee of Tangdu Hospital, Air Force Military Medical University (Approval No. K202407-19) on July 3, 2024, and was registered with the Chinese Clinical Trial Registry (Registration No. ChiCTR2400086596) on July 6, 2024. Patients who underwent thoracoscopic pulmonary resection at Tangdu Hospital between August 2024 and July 2025 were enrolled, with the final follow-ups completed by October 2025. This prediction model study adheres to the Transparent Reporting of a Multivariable Prediction Model for Individual Prognosis or Diagnosis statement. The completed checklist is provided in the supplementary material.

The inclusion criteria were as follows: patients scheduled to undergo thoracoscopic pulmonary resection during the study period (including wedge resection, segmentectomy, or lobectomy); patients who received ultrasound-guided thoracic paravertebral nerve block combined with general anesthesia during surgery; age ≥18 years; ability to communicate via telephone; provision of informed consent and voluntary enrollment in the study; and use of patient-controlled intravenous analgesia (PCIA) after the operation.

The exclusion criteria were as follows: patients who refused to participate in the study; patients with communication disorders or inability to cooperate with the researchers (including language comprehension disorders, mental illness, epilepsy, history of Parkinson's disease, or myasthenia gravis); patients who underwent additional pulmonary or other surgeries within 3 months after the index surgery; patients lost to follow-up due to death, unwillingness to accept telephone follow-up, or other reasons; patients with any preoperative acute or chronic pain-related conditions; and patients whose procedure was converted from VATS to open thoracotomy.

### 2.2 Clinical management protocol

Patients were evaluated before surgery. Upon entering the operating room, continuous monitoring of electrocardiogram, pulse oximetry, and arterial blood pressure was initiated. Anesthesia induction was performed using midazolam 0.03 mg/kg, propofol 2 mg/kg, sufentanil 0.5 μg/kg, and rocuronium 50 mg. After anesthesia induction, an ultrasound-guided thoracic paravertebral block was performed using 20 mL of 0.25% ropivacaine at a single level. Anesthesia was maintained using a combination of intravenous and inhalational anesthetics, including remifentanil at a dosage of 0.05–2 μg/kg/min, dexmedetomidine at 0.2–0.4 μg/kg/h, and sevoflurane at 0.5–2%. Rocuronium bromide was administered as intermittent boluses as needed. The tracheal tube was removed as soon as the patient met extubation criteria. Postoperative analgesia was provided via a PCIA pump for the first 48 hours. The PCIA protocol was as follows: 100 mL solution containing sufentanil 100 μg and tropisetron 5 mg, with a continuous infusion rate of 2.5 mL/h, a bolus dose of 3 mL, and a lockout interval of 15 min. Rescue analgesia was administered on up to three postoperative occasions using flurbiprofen axetil 50 mg. Patients without complete anesthesia records or those who received additional anesthetics for induction or maintenance were excluded.

### 2.3 Outcome measures and relevant definitions

Preoperative data: age, sex, American Society of Anesthesiologists (ASA) physical status classification, body mass index (BMI), smoking and alcohol history, preoperative pain status, duration of illness, and lesion location.

Intraoperative data: extent of resected lung tissue, lung nodule diameter, pathological type, operative time, anesthesia time, intraoperative fluid balance (including blood loss, transfusion volume, fluid intake, and output), use of other medications, and intraoperative blood pressure fluctuations.

Postoperative data: pain scores at rest and during movement (coughing) assessed using the Numerical Rating Scale (NRS) at 3 months postoperatively; the highest acute postoperative pain score within 72 hours; consumption of rescue opioid medication in morphine milligram equivalents (MME) on postoperative days 1–7; incidence of postoperative adverse reactions; postoperative pulmonary complications (assessed via CT scans within 7 days postoperatively); length of postoperative hospital stay; number and duration of chest tube drainage; and duration of stay in the post-anesthesia care unit (PACU).

For postoperative pneumonia, we adopted the standardized diagnostic criteria of the Society of Thoracic Surgeons/European Society of Thoracic Surgeons: radiographic evidence of new or progressive pulmonary infiltrates on chest CT performed within 7 days post-surgery, combined with at least one of the following clinical signs of infection—fever >38 °C, leukocytosis (>12,000/μL) or leukopenia (<4,000/μL), or purulent sputum—and evidence of respiratory dysfunction (hypoxemia defined as SpO_2_ <90% on room air or PaO_2_ <60 mmHg, or increased work of breathing requiring respiratory support) (10). Cases meeting radiographic criteria but lacking clinical or respiratory criteria were classified as radiographic infiltrates rather than clinical pneumonia.

For hydropneumothorax, we defined it as the simultaneous presence of air and pleural effusion on postoperative chest CT, regardless of whether chest tube reinsertion was performed. Most cases were small in volume, asymptomatic, and resolved spontaneously without intervention (11).

Postoperative acute pain was assessed using the NRS as a continuous measure. Mild pain was defined as an NRS score of 0–3, and moderate pain as an NRS score of 4–6. The data collection time points for postoperative acute pain were strictly designed according to the 72-h critical therapeutic window recommended for acute pain treatment. This protocol applied high-frequency dynamic evaluation, with assessments performed at 6, 24, 48, and 72 h after surgery to capture the key stages of pain change (12).

### 2.4 Postoperative follow-up

Two trained investigators conducted one-on-one telephone questionnaires with enrolled patients. The investigators used the NRS to assess whether patients developed postoperative acute pain and CPSP. In addition, they followed up to determine the incidence of postoperative adverse events and pulmonary complications. Specific follow-up questions included: “Do you currently have pain related to your surgery?” As defined by the International Association for the Study of Pain, if the patient's answer was “yes” and other potential causes of pain were ruled out, the patient was considered to have CPSP. In such cases, the investigators proceeded to ask: “Please rate your average pain over the past week on a scale of 0–10 (0 = no pain, 10 = worst pain imaginable)”; “Have you noticed any factors that exacerbate or relieve your pain?”; “Does your surgical site exhibit any signs of infection, such as redness, swelling, or fever?”; and “Since your surgery, have you had any periods of pain related to the surgical site that have come and gone?” To qualify as CPSP, patients were required to report pain localized to the surgical site, the ipsilateral chest wall, the incision(s), or the chest tube drainage site. Pain referred to distant areas without a clear relationship to the surgical field was excluded.

### 2.5 Statistical analysis

Based on previously reported data, an odds ratio (OR) of 2.126 and an exposure prevalence (P2) of 0.494 in the non-CPSP group were assumed. Accounting for an estimated dropout rate of 30%, the PASS software calculated that a minimum sample size of 432 patients would be required (13).

This study employed a paper-based data collection method for data management. Data were entered into Excel using a double-entry process. After the comparison was completed, the data manager used SPSS Statistics version 27.0 for Windows to verify the data. Any discrepancies or issues identified during the verification process, as well as problems encountered during data entry, were summarized in a query log and submitted to the research team for resolution until all data-related problems were addressed. The following methods were used to handle missing data. First, the mechanism of missing data was assessed using Little's MCAR test, confirming that the missingness was completely at random. For variables with a missing proportion of <5%, complete-case analysis was applied. Concurrently, samples with >20% missingness in critical outcome variables were systematically excluded. For isolated missing values in continuous variables, mean imputation was performed. For categorical variables, mode imputation was used, replacing missing values with the most frequent category. For count variables, median imputation was performed to account for skewed distributions. All statistical analyses were conducted separately on both the complete-case and imputed datasets, and the consistency of the results confirmed the reliability of the conclusions.

All statistical tests were two-sided, and a *P*-value <0.05 was considered statistically significant. The Kolmogorov–Smirnov test was used to assess the normality of continuous variables. Normally distributed data were expressed as mean ± standard deviation, and inter-group comparisons were performed using Student's t-test. Non-normally distributed data were expressed as median (interquartile range), and group comparisons were conducted using the Mann–Whitney U-test. Categorical data were expressed as frequency (percentage), and inter-group comparisons were performed using the chi-square test or Fisher's exact test. Variables with a *P*-value <0.1 in univariate analysis were subsequently entered into multivariate logistic regression analysis to identify independent predictors associated with chronic pain after thoracoscopic surgery. A risk prediction model was established using logistic regression in R version 4.1.2 (pROC package) to evaluate the predictive performance for CPSP.

## 3 Results

### 3.1 General data analysis

**Figure 1:**
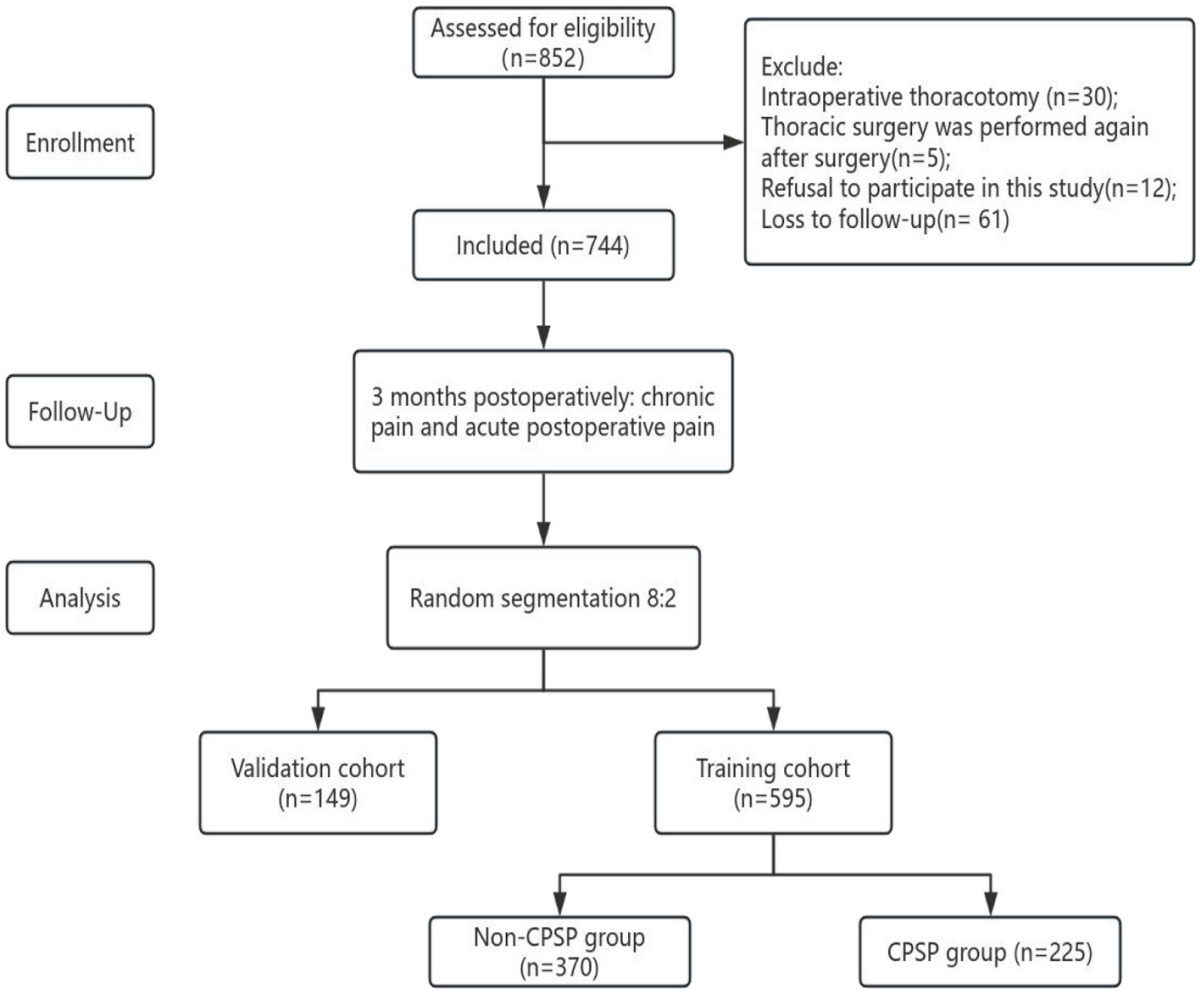
Study flow.

A total of 852 patients were initially screened, of whom 47 did not meet the inclusion criteria; thus, 805 patients were enrolled in the trial. During follow-up, 61 patients were lost to follow-up. The remaining 744 patients provided written informed consent and were included in the final analysis (Figure 1). Baseline characteristics were comparable between patients who completed the study and those who dropped out (Supplementary Table 1).

Among the 744 patients included in the final analysis, 284 (38.1%) developed CPSP. The incidence rates in the training and validation cohorts were 37.8% (225/595) and 36.9% (55/149), respectively, with no statistically significant difference between the two cohorts. In the training cohort, among the 595 patients who completed follow-up after thoracoscopic lung resection, 225 (37.8%) developed chronic pain. Of these, 189 (84.0%) experienced mild pain (NRS 0–3), while 36 (16.0%) experienced moderate-to-severe pain (NRS ≥4). Univariate analysis revealed that chronic pain after thoracoscopic lung resection was significantly associated with gender, diabetes, duration of chest tube drainage, duration of disease, postoperative acute pain, postoperative pneumonia, hydropneumothorax, and opioid rescue dose (all *P* < 0.05) (Tables 1 and 2).

**Table 1:** Baseline demographic and clinical characteristics of the training cohort.

General information	Non-CPSP (*n* = 370)	CPSP (*n* = 225)	*χ^2^/Z*	*P*
Age (y)	60 (51, 66)	58 (51, 65)	−0.951	0.342
Gender, *n* (%)				
Male	200 (54.05)	92 (40.89)	9.703	0.002
Female	170 (45.95)	133 (59.11)		
BMI (kg/m^2^)	23.88 (21.8, 26.0)	23.51 (21.6, 25.4)	−1.325	0.185
Smoking history, *n* (%)				
Non-smoker	90 (24.32)	45 (20.00)	4.011	0.405
Ex-smoker	192 (51.89)	135 (60.00)		
Smoker	85 (23.79)	48 (20.00)		
Alcohol consumption history, *n* (%)				
Non-drinker	116 (31.35)	60 (26.67)	3.574	0.467
Ex-drinker	234 (63.24)	157 (69.77)		
Drinker	17 (5.41)	11 (3.56)		
Hypertension, *n* (%)				
No	252 (68.11)	162 (72.00)	3.907	0.272
Grade I	66 (17.84)	27 (12.00)		
Grade II	27 (7.30)	17 (7.56)		
Grade III	25 (6.76)	19 (8.44)		
Diabetes, *n* (%)	85 (22.97)	31 (13.78)	8.459	0.015

CPSP, Chronic postoperative pain; BMI, Body mass index.

**Table 2:** Disease-related information of the training cohort.

General information	Non-CPSP (*n* = 370)	CPSP (*n* = 225)	χ^2^/*Z*	*P*
Duration of disease (month)	3 (1.0, 12.0)	4 (1.0, 12.0)	−2.157	0.031
Excision extent, *n* (%)				
Left lung	161 (43.51)	101 (44.89)	0.243	0.886
Right lung	208 (56.22)	123 (54.67)		
Whole lung	1 (0.27)	1 (0.44)		
Lesion diameter (cm)	1.5 (1.0, 2.5)	1.5 (1.0, 2.5)	−0.394	0.693
Extent of resected lung tissue, *n* (%)				
Lobectomy	186 (50.27)	115 (51.11)	5.308	0.070
Segmentectomy	59 (15.95)	50 (22.22)		
Pulmonary wedge resection	125 (33.78)	60 (26.67)		
Type of pathology, *n* (%)				
Malignancy	309 (83.51)	182 (80.89)	0.668	0.414
Benign tumor	61 (16.49)	43 (19.11)		
Operation mode, *n* (%)				
Single-port	158 (42.70)	102 (45.33)	0.487	0.784
Two-port	114 (30.81)	64 (28.44)		
Three-port	98 (26.49)	59 (26.22)		
Number of postoperative drainage tubes (counts)	2 (1, 2)	2 (1, 2)	−0.908	0.364
Duration of chest tube drainage (days)	4 (3, 5)	4 (3, 6)	−3.286	0.001
Opioid rescue dose (MME)	40 (20, 60)	50 (30, 70)	−5.672	<0.001
Operation duration (min)	99.5 (70.0, 130.0)	104.0 (75.0, 130.0)	−0.684	0.494
Anesthesia duration (min)	125.0 (90.0, 155.5)	128.0 (95.0, 154.5)	−0.596	0.551
PACU time (min)	40 (30.0, 56.0)	40 (30.0, 60.0)	−0.151	0.880
ASA classification, n (%)				
Grade I	31 (8.38)	9 (4.00)	4.285	0.117
Grade II	318 (85.95)	203 (90.22)		
Grade III	21 (5.68)	13 (5.78)		
Blood loss (ml)	30 (20, 50)	30 (20, 50)	−1.119	0.263
Pulmonary complications, *n* (%)				
Hydropneumothorax	173 (46.76)	143 (63.56)	15.855	<0.001
Pneumonia	194 (52.43)	140 (62.22)	5.446	0.020
Pleural effusion	112 (30.27)	79 (35.11)	1.504	0.220
Atelectasis	110 (29.73)	57 (25.33)	1.339	0.247
Subcutaneous emphysema	210 (56.76)	141 (62.67)	2.020	0.155
Postoperative adverse reactions, *n* (%)				
Nausea/vomiting	50 (13.5)	27 (12.0)	0.284	0.594
Dizziness	16 (4.3)	10 (4.4)	0.005	0.945
Pruritus	9 (2.4)	5 (2.2)	0.027	0.870
Respiratory depression	0 (0)	0 (0)	0.000	1,000
Postoperative acute pain (scores)	3 (2.0, 4.3)	7 (5.0, 9.0)	−14.786	<0.001

MME, morphine milligram equivalents; PACU, post-anesthesia care unit; ASA, American society of anesthesiologists.

### 3.2 Multivariate analysis

Based on the data from the training set, multivariate logistic regression analysis showed that postoperative acute pain (OR = 2.148, 95% CI: 1.895–2.435, *P* < 0.001), female sex (OR = 1.907, 95% CI: 1.203–3.023, *P* = 0.006), postoperative pneumonia (OR = 1.963, 95% CI: 1.235–3.120, *P* = 0.004), duration of chest tube drainage (OR = 1.143, 95% CI: 1.025–1.274, *P* = 0.017), and opioid rescue dose (OR = 1.009, 95% CI: 1.002–1.016, *P* = 0.011) were significant independent predictors associated with the development of chronic pain after thoracoscopic surgery (Table 3). Subgroup analysis stratified by the extent of lung resection (lobectomy, segmentectomy, and wedge resection) confirmed that the model maintained good discriminative ability across all three surgical extents, with AUC values ranging from 0.866 to 0.896 (Supplementary Table 3).

**Table 3:** CPSP multi-factor logistic regression analysis.

	OR	95% CI	Wald *χ2*	*P*
Female	1.948	1.222–3.106	7.848	0.005
Duration of chest tube drainage	1.138	1.020–1.270	5.335	0.021
Opioid rescue dose	1.008	1.002–1.015	5.782	0.016
Postoperative pneumonia	1.953	1.228–3.106	8.006	0.005
Postoperative acute pain	2.154	1.899–2.443	142.646	<0.001
Diabetes	1.834	0.968–3.476	3.460	0.063
Hydropneumothorax	1.110	0.700–1.758	0.197	0.657
Duration of disease	0.997	0.984–1.011	0.163	0.686
Extent of resected lung tissue	0.923	0.708–1.202	0.355	0.551

OR, Odds ratio; CI, Confidence interval.

### 3.3 Predictive model construction

#### 3.3.1 Nomogram

**Figure 2:**
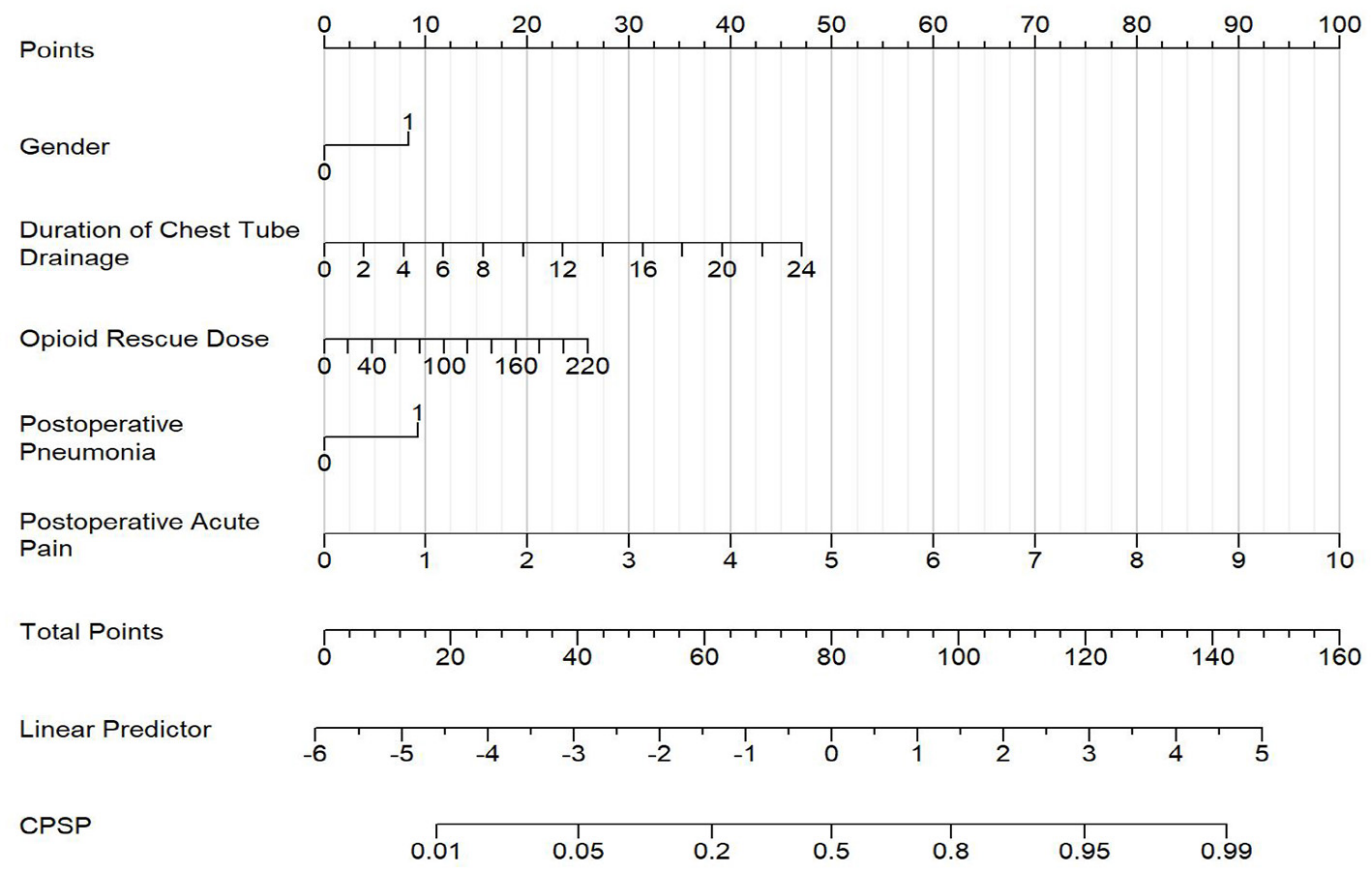
The prediction model of chronic postsurgical pain (Gender: 0 = Male, 1 = Female;Postoperative pneumonia: 0 = No, 1 = Yes): to use the nomogram, locate each patient's value on the corresponding predictor axis, draw a vertical line upward to the “Points” scale to obtain individual points, sum all points to obtain the total score, and then draw a vertical line downward from the total score axis to the “Risk of CPSP” axis to estimate the probability of developing chronic pain at 3 months post-surgery. For example, a patient with a total score >60 (corresponding to risk >50%) may be considered for intensified multimodal analgesic interventions.

A nomogram for predicting the risk of chronic pain following thoracoscopic lung resection was developed based on the training cohort and is presented in Figure 2. This tool incorporates the following key predictors: gender (1 = female, 0 = male), duration of chest tube drainage, opioid rescue dose, postoperative pneumonia (1 = yes, 0 = no), and postoperative acute pain. On the predictor axis, each variable is assigned a scale range based on its contribution, with higher β coefficients corresponding to higher point scores.

#### 3.3.2 Calibration curves

**Figure 3:**
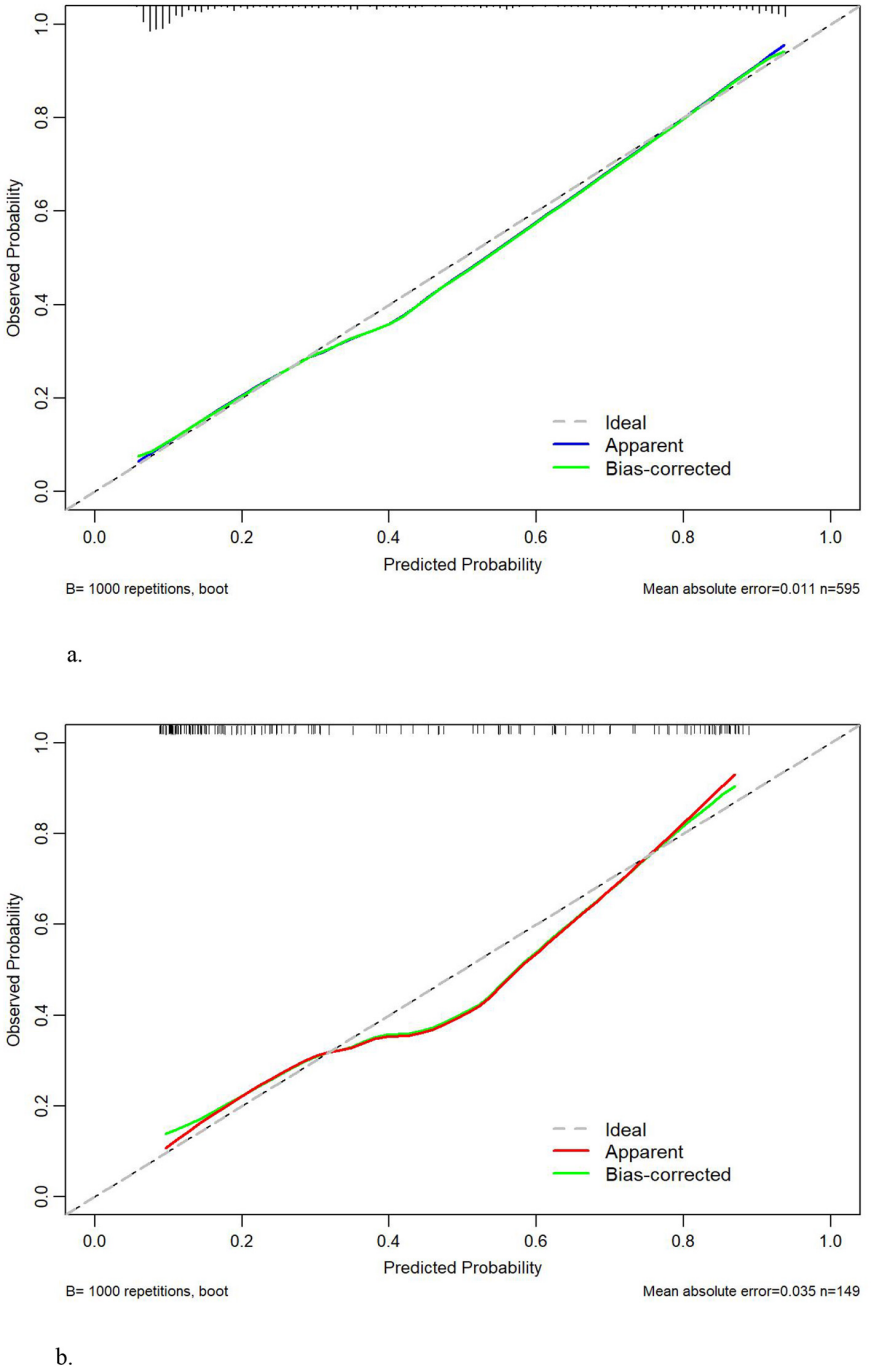
(**a**) Calibration curve of the nomogram for predicting CPSP in the training cohort. (**b**) Calibration curve of the nomogram for predicting CPSP in the validation cohort.

Calibration curves were generated to assess the agreement between predicted probabilities and observed outcomes for both cohorts (Figures 3a, b). The calibration curves closely approximated the ideal diagonal line, indicating good model fit. The Hosmer–Lemeshow test yielded non-significant results for both the training set (*P* = 0.737) and the validation set (*P* = 0.765), confirming no significant deviation between predicted and observed frequencies. Additionally, calibration was assessed using the calibration slope and intercept. In the training cohort, the calibration slope was 1.00 (95% CI: 0.85–1.15) and the intercept was −0.00 (95% CI: −0.23–0.23). In the validation cohort, the calibration slope was 0.76 (95% CI: 0.51–1.02) and the intercept was −0.11 (95% CI: −0.52 to 0.30). These values indicate no significant miscalibration. Collectively, these findings demonstrate excellent calibration accuracy of the nomogram for clinical prediction.

#### 3.3.3 Receiver-operating characteristics (ROC) analysis

**Figure 4:**
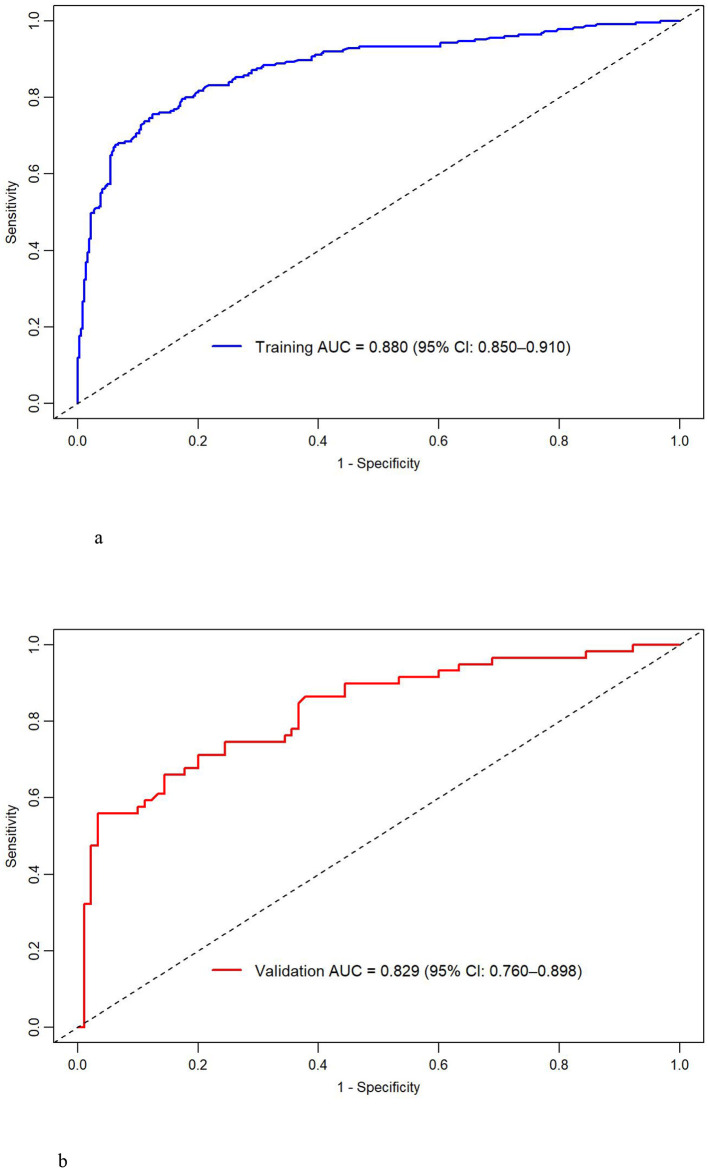
(**a**) ROC curve of chronic postsurgical pain prediction model in the training cohort. (**b**) ROC curve of chronic postsurgical pain prediction model in the validation cohort.

The predictive model, established based on postoperative acute pain assessment, demonstrated excellent discrimination and calibration. The calibration curve closely approximated the ideal diagonal line, indicating satisfactory calibration. The model's area under the curve (AUC) was 0.880 (95% CI: 0.850–0.910) in the training set and 0.829 (95% CI: 0.760–0.898) in the validation set (Figures 4a, b).

#### 3.3.4 Decision curve analysis (DCA)

**Figure 5:**
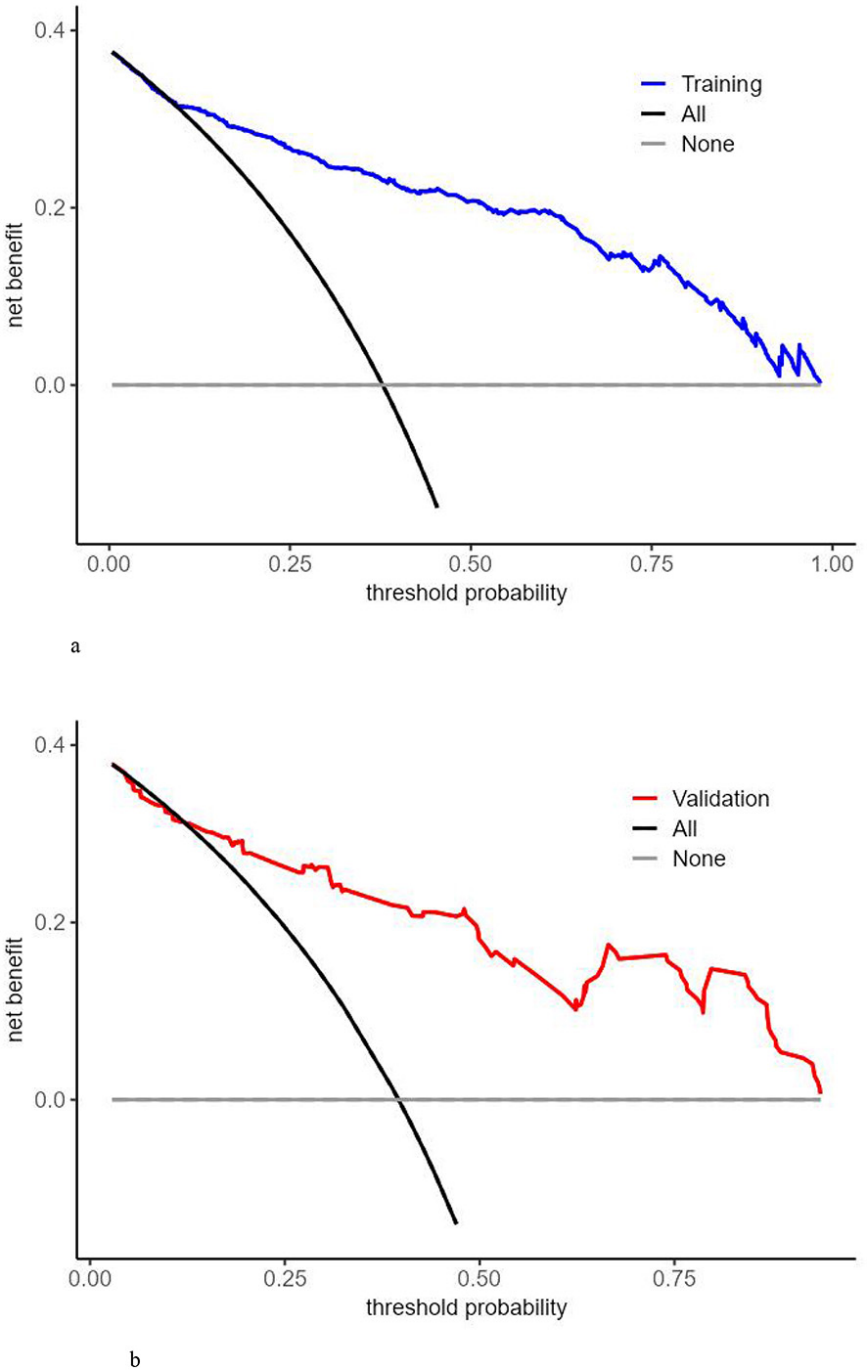
(**a**) Decision curve analysis of chronic postsurgical pain prediction model in the training cohort. (**b**) Decision curve analysis of chronic postsurgical pain prediction model in the validation cohort.

DCA quantifies the net benefit across various decision thresholds to guide clinical decision-making. In the training cohort, the prediction model demonstrated a positive net benefit across a wide range of clinically reasonable threshold probabilities, particularly between 10 and 60%, indicating its utility for informing clinical decisions. In the validation cohort, the model maintained a favorable net benefit compared with default strategies (treat-all or treat-none) within the threshold probability range of 20–60%, suggesting robust performance in supporting individualized risk stratification. Although the net benefit decreased at higher thresholds, the model still outperformed the default strategies within certain intervals (Figures 5a, b).

#### 3.3.5 Model validation

To further evaluate model performance, we calculated confusion matrices and Brier scores for the validation cohort. The confusion matrix showed that the model achieved a sensitivity of 70.9% (39/55) and a specificity of 78.7% (74/94). The corresponding false negative rate was 29.1% (16/55), and the false positive rate was 21.3% (20/94) (Supplementary Figure 1). The Brier score was 0.1605 (95% CI: 0.132–0.189), indicating a small average error between predicted probabilities and actual outcomes, reflecting acceptable calibration.

## 4 Discussion

This study analyzed the incidence and associated predictors of chronic pain in 744 patients who underwent thoracoscopic lung resection and constructed a risk prediction model for chronic pain at 3 months post-surgery. The incidence of CPSP was 38.1%, with the majority of patients (84.5%) experiencing mild and tolerable pain, while a smaller proportion (15.5%) experienced moderate-to-severe pain. Multivariate logistic regression analysis identified gender, postoperative acute pain, postoperative pneumonia, duration of chest tube drainage, and opioid rescue dose as independent predictors associated with CPSP.

The results of this study showed that the proportion of patients with moderate to severe acute pain within 1 week after surgery was significantly higher in the CPSP group than in the non-CPSP group. This finding highlights that poor control of postoperative acute pain is an important risk factor for the onset of CPSP, which is consistent with the findings of Fiorelli et al. (8). Acute pain after surgery occurs immediately following the operation and usually lasts no more than 3–7 days. During this period, the severity and duration of acute postoperative pain, as well as the increase in analgesic consumption, have all been identified as reliable predictors of CPSP development—a relationship consistently demonstrated in multiple prospective cohort studies (14, 15). If poorly managed in the early stages, prolonged and repeated pain stimulation may trigger central and peripheral sensitization, potentially evolving into CPSP after three months (16). Therefore, clinicians should pay greater attention to the early identification and prevention of the transition from postoperative acute pain to chronic pain in high-risk patients to optimize acute pain management. It has been proposed that perioperative multimodal analgesia, along with continuous follow-up and treatment after discharge, represents an important strategy for identifying and managing the transformation of acute pain into chronic pain (17). Our results indicate that patients with poor acute postoperative pain control, particularly those with moderate to severe pain, are at a significantly higher risk of developing CPSP compared to those with well-controlled pain. Nevertheless, this finding requires confirmation in large-sample prospective studies.

Numerous studies indicate that female patients experience higher rates of both chronic pain and neuropathic pain following VATS compared to males (8). We also identified female sex as an independent risk factor for thoracoscopic CPSP. This may result from the interaction of physiological and psychological factors or be related to differences in brain structure and function. Consequently, the peak incidence of CPSP in women occurs during perimenopause (ages 45–55), followed by a decline with advancing age (18). Additionally, women may be more susceptible to emotional influences, and significant mood fluctuations can reduce pain tolerance.

Postoperative thoracic complications contribute to the development of CPSP, and we observed that the presence of pneumonia within 3 months after surgery was an independent risk factor for CPSP. Ulger et al. reported that postoperative increases in monocyte and neutrophil counts were positively correlated with acute pain scores (19). To date, no studies have explicitly elucidated the specific relationship between postoperative pneumonia and chronic pain, which may be attributable to the persistent release of inflammatory factors associated with prolonged postoperative pneumonia. Inflammatory responses are triggered and propagated by cytokines, which play a key role in the development of both acute pain and CPSP. Pro-inflammatory cytokines are important in upregulating the inflammatory response, and if not properly controlled, this can lead to persistent pain and impaired tissue healing (20).

Furthermore, our study indicates that prolonged chest tube drainage after VATS is associated with extended hospital stays, which consequently delays physical recovery and imposes additional financial and psychological burdens on patients. Studies have shown that there may be an association between the total volume of pleural effusion drained and the development of CPSP in patients undergoing thoracoscopic lung resection, although the specific causal relationship and underlying mechanisms remain to be fully elucidated (21). When the perioperative pleural effusion volume is ≤ 400 mL and there is no air leakage, early and rapid removal of the chest tube can effectively alleviate postoperative pain and improve lung function (22). Chen et al. (23) demonstrated that the use of narrow-bore chest tubes for postoperative drainage significantly reduces the incidence of CPSP and promotes better wound healing. In addition, a large volume of pleural effusion can cause pleural inflammation and adhesion, which not only affects normal lung expansion but may also further stimulate or compress surrounding nerves, thereby contributing to the onset of CPSP.

At present, research on the development of risk prediction models for chronic pain after thoracoscopy remains scarce. Reported incidence rates of CPSP vary across studies owing to differences in follow-up time points and pain assessment tools, leading to a lack of consistency among existing prediction models. In this context, our study used R programming to build a comprehensive prediction model for CPSP, incorporating a nomogram, calibration curve, ROC curve, and DCA. The model underwent a rigorous development and validation process: predictor selection and model construction were first performed using the training set (80% of the cohort), followed by performance evaluation on the internal validation set (20%). The model demonstrated good fit and was able to provide personalized risk assessments based on individual patient characteristics. A meta-analysis of existing prediction models for surgical CPSP (e.g., in breast surgery, orthopedic surgery, and hernia repair) reported that the most common predictors included preoperative pain, age, gender, and postoperative acute pain (24). Chen et al. selected four variables based on LASSO regression—body mass index, chronic pain history, miR550a-3p, and visual analog scale score on the second postoperative day—to build a nomogram for predicting CPSP risk after VATS in patients with lung adenocarcinoma (25). There is substantial overlap between the predictors identified in these models and those in our study, indicating that these factors play an important role in the development and progression of CPSP. Therefore, for high-risk populations, early prevention and treatment of acute pain should be a priority.

In addition to constructing a nomogram, our study performed a multi-dimensional validation of the model, including calibration curves and ROC curves, to comprehensively evaluate its predictive accuracy and overall performance. We also conducted decision curve analysis (DCA) to assess the net clinical benefit of the model. This comprehensive evaluation framework accounts for clinical uncertainties and individual patient differences, thereby helping to avoid the potential problems of overdiagnosis or undertreatment that may arise from using nomograms alone. Based on the model's risk stratification scores, patients can be classified into high-risk (score ≥80%), intermediate-risk (20–80%), and low-risk (<20%) groups. The model may assist clinicians in identifying patients at higher risk of CPSP, who could then be prioritized for closer postoperative follow-up (e.g., telephone monitoring at 2 and 4 weeks after discharge) or considered for enrollment in future trials of targeted preventive interventions. However, these potential applications require prospective validation before any clinical recommendations can be made.

There are several limitations to this study. First, the assessment of postoperative acute pain, while clinically relevant, may benefit from more objective or frequent measures in future research. Future prospective studies should validate these assessments using approaches such as mobile digital pain scales for real-time recording or multimodal data collection methods, including the monitoring of heart rate variability at key time points (e.g., 24, 48, and 72 h after surgery). In addition, memory aids such as “pain calendars” could be used to reduce recall bias. Second, although internal validation demonstrated good predictive performance of the model, its generalizability remains limited owing to the single-center design. Future multicenter studies with larger sample sizes should perform external validation and, ideally, bootstrap or cross-validation to obtain optimism-corrected estimates, along with subgroup analyses or formal tests for interaction between resection type and model performance, before the model can be recommended for routine clinical use. Third, we did not assess neuropathic pain characteristics (e.g., using the DN4 questionnaire, PainDETECT, or any structured neuropathic pain assessment tool); therefore, we could not distinguish neuropathic from nociceptive components of CPSP. Furthermore, although baseline demographic characteristics and clinical parameters were collected, we failed to systematically assess psychological predictors of CPSP, including anxiety, depression, and pain catastrophizing. Future prospective studies should incorporate psychological assessment tools to further explore the mechanisms by which psychosocial factors contribute to the development of chronic postoperative pain. Finally, CPSP was assessed at a single time point (3 months post-surgery), which may not capture intermittent or recurrent pain episodes and could potentially underestimate the true burden of CPSP. Patients with fluctuating pain trajectories who were pain-free at the assessment point may have been misclassified as non-CPSP. Future studies should incorporate multiple longitudinal assessments (e.g., at 3, 6, and 12 months) and consider using pain diaries or ecological momentary assessment to better characterize the dynamic nature of chronic postsurgical pain.

## 5 Conclusion

In patients undergoing thoracoscopic pulmonary resection, female sex, prolonged chest tube drainage, higher opioid rescue doses, higher postoperative acute pain scores, and postoperative pneumonia are independent predictors associated with the development of chronic postsurgical pain at 3 months. The proposed nomogram provides good risk stratification and may help clinicians identify high-risk individuals for closer postoperative monitoring. However, whether risk-stratified interventions can reduce CPSP requires prospective validation.

## Data Availability

The raw data supporting the conclusions of this article will be made available by the authors, without undue reservation.
