# Supplementary material for: Development and validation of a predictive model for chronic pain after thoracoscopic pulmonary resection
Source: Front Public Health. 2026 Jun 19;14:1787875. doi: 10.3389/fpubh.2026.1787875 (PMC13328369; doi:10.3389/fpubh.2026.1787875)
Supplement: Supplementary file 1 [file Data_Sheet_1.docx]

**Supplementary Material 3:**

**Detailed Reproducibility Information for the Prediction Model Logistic Regression Equation:**

**1. The probability (p) of developing chronic postsurgical pain (CPSP) is calculated as:**Logit(p) = ln(p / (1-p)) = –5.378 + (0.667 × Gender) + (0.129 × Duration of chest tube drainage) + (0.008 × Opioid rescue dose) + (0.670 × Postoperative pneumonia) + (0.767 × Postoperative acute pain)

1. **Model Intercept and Coefficients (from the revised multivariate model):**

| Predictor | Coding | Coefficient (B) | Intercept |
| --- | --- | --- | --- |
| Intercept | – | – | - 5.378 |
| Gender | 1 = Female, 0 = Male | 0.667 | – |
| Duration of chest tube drainage | continuous (days) | 0.129 | – |
| Opioid rescue dose | continuous (MME) | 0.008 | – |
| Postoperative pneumonia | 1 = Yes, 0 = No | 0.670 | – |
| Postoperative acute pain | continuous (NRS 0–10) | 0.767 |  |

1. **Worked Example:**
   **Patient characteristics:** Gender: Female → Gender_female = 1; Duration of chest tube drainage: 5 days; Opioid rescue dose: 50 MME; Postoperative pneumonia: No → Pneumonia = 0; Postoperative acute pain (highest NRS): 8

**Step 1 – Calculate Logit(p):**

Logit(p) = –5.378 + (0.667 × 1) + (0.129 × 5) + (0.008 × 50) + (0.670 × 0) + (0.767 × 8) = –5.378 + 0.667 + 0.645 + 0.4 + 0 + 6.136 = 2.470

**Step 2 – Calculate probability (p):**

p = 1 / (1 + e^{–2.470}) = 1 / (1 + 0.0846) = 1 / 1.0846 ≈ 0.922

**Interpretation:**

This female patient has an estimated 92.2% probability of developing chronic postsurgical pain at 3 months after thoracoscopic pulmonary resection.
