# Supplementary material for: Development and validation of a predictive model for chronic pain after thoracoscopic pulmonary resection
Source: Front Public Health. 2026 Jun 19;14:1787875. doi: 10.3389/fpubh.2026.1787875 (PMC13328369; doi:10.3389/fpubh.2026.1787875)
Supplement: Supplementary file 2 [file Table_1.docx]

**Supplementary Table**

Supplementary Table 1 Comparison of baseline characteristics between included and excluded patients

| ***General information*** | ***Included patients (n=744)*** | ***excluded patients (n=108)*** | *χ^2^/* ***Z*** | ***P*** |
| --- | --- | --- | --- | --- |
| Age (y) | 59 (51, 66) | 59 (48, 65) | -0.370 | 0.711 |
| Gender, n(%) |  |  | 0.232 | 0.630 |
| Male | 356 (47.8) | 49 (45.4) |  |  |
| Female | 388 (52.8) | 59 (54.6) |  |  |
| BMI (kg/m^2^) | 23.67 (21.63, 25.71) | 24.02 (22.09, 25.87) | -0.833 | 0.405 |
| ASA classification, n(%) |  |  | 0.110 | 0.947 |
| Grade I | 49 (6.6) | 8 (7.4) |  |  |
| Grade II | 648 (87.1) | 93 (86.1) |  |  |
| Grade III | 47 (6.3) | 7 (6.5) |  |  |

CPSP: Chronic postoperative pain; BMI: Body mass index; ASA: American Society of Anesthesiologists

Supplementary Table 2 Baseline demographic and disease-related information of the validation cohort

| ***General information*** | ***Non-CPSP (n=90)*** | ***CPSP (n=59)*** | *χ^2^/* ***Z*** | ***P*** |
| --- | --- | --- | --- | --- |
| Age (y) | 60.000 (52.0, 64.0) | 59.000 (51.0, 65.0) | -0.515 | 0.607 |
| Gender, n (%) |  |  | 0.197 | 0.657 |
| Male | 43 (47.78) | 26 (44.07) |  |  |
| Female | 47 (52.22) | 33 (55.93) |  |  |
| BMI (kg/m^2^) | 23.19±2.88 | 24.29±3.60 | -2.053 | 0.042 |
| Hypertension, n(%) |  |  | 5.170 | 0.160 |
| No | 52 (57.78) | 39 (66.10) |  |  |
| Grade I | 20 (22.22) | 6 (10.17) |  |  |
| Grade II | 15 (16.67) | 9 (15.25) |  |  |
| Grade III | 3 (3.33) | 5 (8.47) |  |  |
| Diabetes, n (%) | 19 (21.11) | 7 (11.86) | 2.115 | 0.146 |
| Duration of disease (month) | 4 (1.0, 12.0) | 4 (1.0, 24.0) | -0.745 | 0.456 |
| Excision extent, n (%) |  |  | 0.006 | 0.939 |
| Left lung | 33 (36.67) | 22 (37.29) |  |  |
| Right lung | 57 (63.33) | 37 (62.71) |  |  |
| Lesion diameter (cm) | 1.45 (1.0, 2.1) | 1.50 (1.0, 2.1) | -0.117 | 0.907 |
| Extent of resected lung tissue, n (%) |  |  | 3.955 | 0.138 |
| Lobectomy | 41 (45.56) | 35 (59.32) |  |  |
| Segmentectomy | 19 (21.11) | 6 (10.17) |  |  |
| Pulmonary wedge resection | 30 (33.33) | 18 (30.51) |  |  |
| Type of pathology, n (%) |  |  | 1.008 | 0.315 |
| Malignancy | 76 (84.44) | 46 (77.97) |  |  |
| Benign tumor | 14 (15.56) | 13 (22.03) |  |  |
| Operation Mode, n (%) |  |  | 0.262 | 0.877 |
| Single-port | 30 (33.33) | 22 (37.29) |  |  |
| Two-port | 30 (33.33) | 18 (30.51) |  |  |
| Three-port | 30 (33.33) | 19 (32.20) |  |  |
| Number of postoperative drainage tubes (counts) | 1 (1, 2) | 2 (1, 2) | -0.839 | 0.402 |
| Duration of chest tube drainage (days) | 4 (3, 5) | 3 (3, 6) | -0.238 | 0.812 |
| Opioid rescue dose (MME) | 40 (27.5, 60) | 50 (40, 90) | -2.172 | 0.030* |
| Operation duration (min) | 102.84 ± 44.85 | 97.88 ± 45.63 | 0.656 | 0.513 |
| Anesthesia duration (min） | 126.37 ± 45.05 | 122.03 ± 45.66 | 0.571 | 0.569 |
| **PACU time (min)** | 42.5 (30.0, 60.0) | 40.0 (30.0, 60.0) | -0.035 | 0.972 |
| ASA classification, n (%) |  |  | 0.098 | 0.952 |
| Grade I | 5 (5.56) | 4 (6.78) |  |  |
| Grade II | 77 (85.56) | 50 (84.75) |  |  |
| Grade III | 8 (8.89) | 5 (8.47) |  |  |
| Blood loss (ml) | 30 (20, 50) | 20 (20, 50) | -0.345 | 0.730 |
| **Pulmonary complications, n (%)** |  |  |  |  |
| Hydropneumothorax | **49 (54.44)** | **41 (69.49)** | **3.374** | **0.066** |
| Pneumonia | **47 (52.22)** | **36 (61.02)** | **1.117** | **0.291** |
| Pleural effusion | **32 (35.56)** | **20 (33.90)** | **0.043** | **0.836** |
| Atelectasis | **24 (26.67)** | **15 (25.42)** | **0.028** | **0.866** |
| Subcutaneous emphysema | **51 (56.67)** | **36 (61.02)** | **0.278** | **0.598** |
| Postoperative acute pain (scores) | 3 (2.0, 5) | 7 (5.0, 8.0) | -6.995 | < 0.001 |

CPSP: Chronic postoperative pain; BMI: Body mass index; ASA: American Society of Anesthesiologists; MME: morphine milligram equivalents; PACU: post-anesthesia care unit

**Supplementary Table 3. Subgroup analysis stratified by extent of lung resection**

| ***Subgroup (Resection type)*** | ***AUC (95% CI)*** | ***Predictor*** | ***OR (95% CI)*** | ***P*** |
| --- | --- | --- | --- | --- |
| Lobectomy (n=301) | 0.896 (0.856–0.936) | Gender | 0.697 (0.363 - 1.339) | 0.279 |
|  |  | Duration of chest tube drainage | 1.202 (1.053 - 1.372) | 0.006 |
|  |  | Opioid rescue dose | 1.007 (0.998 - 1.016) | 0.128 |
|  |  | Postoperative pneumonia | 1.706 (0.873 - 3.334) | 0.118 |
|  |  | Postoperative acute pain | 2.219 (1.857 - 2.651) | < 0.001 |
| Segmentectomy (n=109) | 0.887 (0.826–0.948) | Gender | 0.339 (0.110 - 1.040) | 0.059 |
|  |  | Duration of chest tube drainage | 0.755 (0.504 - 1.131) | 0.173 |
|  |  | Opioid rescue dose | 1.018 (0.998 - 1.038) | 0.084 |
|  |  | Postoperative pneumonia | 1.436 (0.501 - 4.121) | 0.501 |
|  |  | Postoperative acute pain | 2.197 (1.638 - 2.946) | < 0.001 |
| Pulmonary wedge resection (n=185) | 0.866 (0.806–0.926) | Gender | 0.454 (0.189 - 1.090) | 0.077 |
|  |  | Duration of chest tube drainage | 1.231 (0.917 - 1.653) | 0.166 |
|  |  | Opioid rescue dose | 1.010 (0.995 - 1.025) | 0.208 |
|  |  | Postoperative pneumonia | 3.033 (1.309 - 7.028) | 0.010 |
|  |  | Postoperative acute pain | 2.053 (1.645 - 2.562) | < 0.001 |

**Interpretation:** The prediction model maintained good discriminative ability across all three resection types, with AUCs ranging from 0.866 to 0.896. Postoperative acute pain was a significant predictor in all subgroups (*P* < 0.001). Duration of chest tube drainage was significant only in the lobectomy subgroup, while postoperative pneumonia reached significance only in the wedge resection subgroup. No formal interaction between resection type and any predictor was statistically significant, indicating overall model robustness.
