# Supplementary figures and images for: Development and validation of a predictive model for chronic pain after thoracoscopic pulmonary resection
Source: Front Public Health. 2026 Jun 19;14:1787875. doi: 10.3389/fpubh.2026.1787875 (PMC13328369; doi:10.3389/fpubh.2026.1787875)

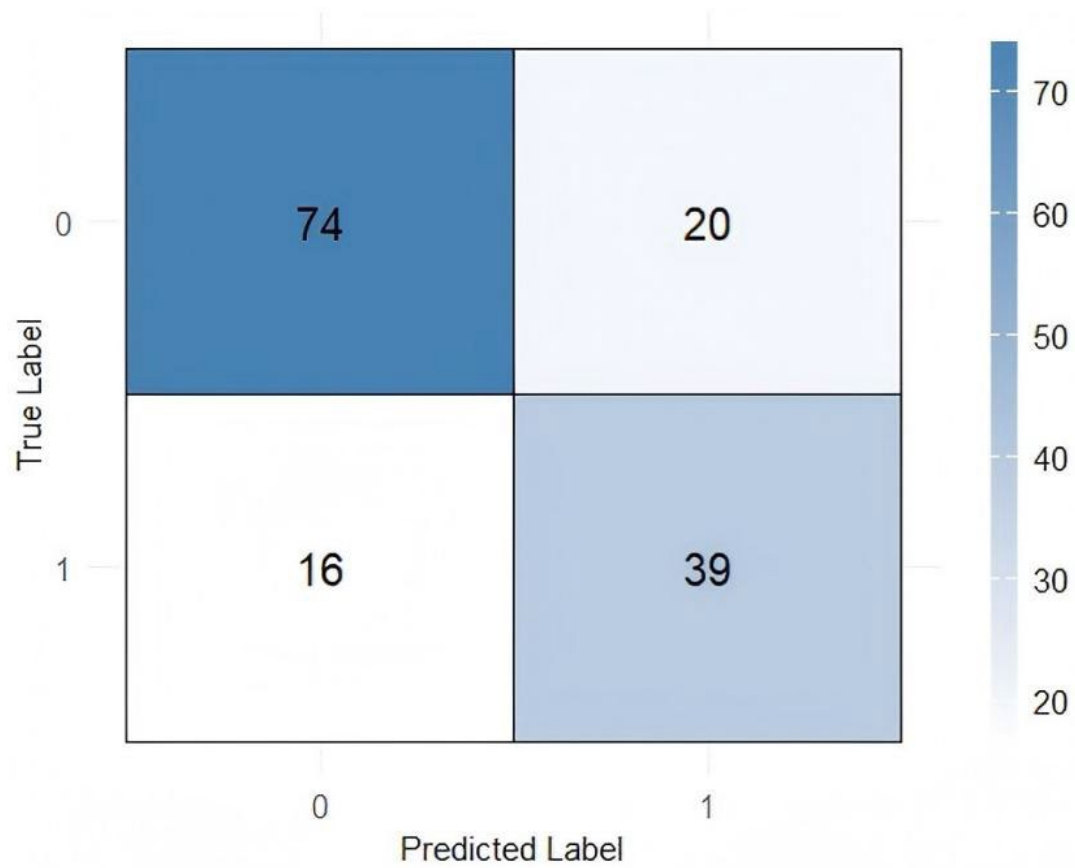

Supplementary Figure 1 Confusion matrix of the prediction model on the validation set

Supplement: Supplementary file 3 [file Image_1.pdf]
